# New Insights into Modelling Bacterial Growth with Reference to the Fish Pathogen *Flavobacterium psychrophilum*

**DOI:** 10.3390/ani10030435

**Published:** 2020-03-05

**Authors:** Christopher D. Powell, Secundino López, James France

**Affiliations:** 1 Department of Animal Biosciences, University of Guelph, Guelph, ON N1G 2W1, Canada; jfrance@uoguelph.ca; 2 Departamento de Producción Animal, Universidad de León, E-24007 León, Spain; 3 Instituto de Ganadería de Montaña, CSIC-Universidad de León, Finca Marzanas s/n, 24346 Grulleros, Spain

**Keywords:** farmed fish, bacterial diseases, *Flavobacterium psychrophilum*, modelling

## Abstract

**Simple Summary:**

*Flavobacteriumpsychrophilum* is a cold-water bacterium responsible for cold water disease and rainbow trout fry syndrome which has significant impacts on fish health and, by extension, negative economic impacts on aquaculture operations. Models can be applied to bacterial growth curves yielding parameter estimates describing rates of bacterial growth and the time it takes for a bacterium to reach its exponential phase of growth (lag time). These parameter estimates can be used to establish the relationship between microbial growth and environmental variables such as pH, temperature and effect of anti-microbial treatments. Two novel models are derived and their potential to describe bacterial growth assessed through their ability to mimic the growth of *Flavobacterium* *psychrophilum* on liquid media. Due to their mechanistic derivation, the proposed models result in flexible and robust growth functions that can be expressed as equations with biologically meaningful parameters. Based upon statistical measures of goodness-of-fit and cross-validation, the purposed models were able to describe satisfactorily the growth of *Flavobacterium* *psychrophilum* on various media. Furthermore, the proposed models also provide insight into underlying mechanisms that are driving microbial growth and how the current environment affects bacterial rate of growth.

**Abstract:**

Two new models, based upon the principles promulgated by Baranyi and co-workers are presented and resulting growth functions evaluated based upon their ability to mimic bacterial growth of the fish pathogen *Flavobacterium psychrophilum*. These growth functions make use of a dampening function to suppress potential growth, represented by a logistic, and are derived from rate:state differential equations. Dampening effects are represented by a rectangular hyperbola or a simple exponential, incorporated into a logistic differential equation and solved analytically resulting in two newly derived growth equations, viz. logistic × hyperbola (log × hyp) and logistic × exponential (log × exp). These characteristics result in flexible and robust growth functions that can be expressed as equations with biologically meaningful parameters. The newly derived functions (log × hyp and log × exp), along with the Baranyi (BAR), simple logistic (LOG) and its modified form (MLOG) were evaluated based upon examination of residuals and measures of goodness-of-fit and cross-validation. Using these criteria, log × hyp, log × exp and BAR performed better than, or at least equally well as, LOG and MLOG. In contrast with log × exp and BAR, log × hyp can be easily manipulated mathematically allowing for simple algebraic expressions for time and microbial biomass at inflexion point, in addition to maximum and scaled maximum growth rates.

## 1. Introduction

With a move towards more exact methods in microbiology, the demand for less empirical models increases [1]. Although many equations are able to describe bacterial growth, to be considered a mathematical model and not just a convenient relationship for empirically fitting to data, an equation should have a sound physiological basis underlying the relationship [1,2,3]. The use of growth functions in the field of microbiology is widespread. In predictive food microbiology, these functions are used in so-called primary modelling to yield useful parameter estimates such as relative growth rate and lag time. These growth features are often then used for further model development (viz. secondary or tertiary modelling) to establish the relationship between microbial growth and environmental variables (e.g., pH, temperature). These estimates of growth parameters enable the prediction of the time required for pathogenic or foodborne microorganisms to reach a critical limit, resulting in clinical signs of disease or food spoilage [4]. Growth functions also find applications in the animal health field. With respect to the field of aquaculture, growth of the pathogenic bacteria *Aeromonas hydrophila* and *Vibrio alginolyticus* has been described using modified versions of the logistic and Gompertz equations [5].

Traditionally, microbial growth measurements have been made using the viable counts method. However, estimating microbial growth parameters from fitting growth functions to optical density measurements has become a more common practice, with the advantages of being rapid and inexpensive compared to the viable counts method [4]. Optical density measurements have been used extensively to describe bacterial growth in various fields of microbiology, in addition to allowing for accurate comparison of model-derived parameter estimates [4,6,7,8,9,10]. Through the correlation between the intensity of light absorbed/reflected and the number of cells in the culture medium, optical density measurements provide an estimate of microbial mass [11]. Regardless of the measure (viable count vs. optical density), microbial growth can be characterized by three distinct phases, viz. the lag, ‘exponential’ and stationary phases. The growth rate of the microbial population increases rapidly until a maximum is reached. The intercept between initial microbial biomass and the tangent to the growth curve at the time where growth rate reaches a maximum is generally referred to as the lag phase. Following the so-called exponential phase, growth rate decreases steadily towards zero reaching a stationary phase, resulting overall in a sigmoidal shape [12].

Microbial growth is commonly described using a number of sigmoidal functions, e.g., the logistic, Gompertz and the purpose-built Baranyi and Huang equations [2,13,14,15,16,17,18,19]. The logistic and the Gompertz equations were initially derived to describe population growth and human mortality, respectively [20,21]. Zwietering et al. [12] modified these functions, plotting the logarithm of the relative population size [ln(*x*/*x*_0_)] against time and attempting to ascribe increased biological meaning to some of the parameters. However, due to these modifications the resulting growth functions are not simple transformations of the original mechanistically-derived Gompertz and logistic equations and are better referred to as modified Gompertz and logistic equations [22]. As a result, mechanistic interpretation is no longer straightforward, and these modified models tend to give empirical descriptions of the sigmoidal pattern of microbial growth. Using a more detailed mechanistic approach, Baranyi and Roberts developed a six-parameter model [23], again plotting log-transformed data [ln(*x*)] against time. However, fitting this particular model can be challenging when using standard nonlinear regression programs [24,25]. Following re-parameterization and incorporation of a number of additional assumptions, a simplified four-parameter model was proposed which is the most widely applied of the Baranyi equations [2,7,15,24].

*Flavobacterium psychrophilum* is a gram-negative yellow-pigmented bacterium and the etiological agent of both cold-water disease (CWD) and rainbow trout fry syndrome (RTFS) [26,27]. *F. psychrophilum* can be found across a wide geographic range covering North and South America, Europe and parts of Asia [28]. Although found primarily in salmonid species, *F. psychrophilum* has been isolated in non-salmonids that inhabit water temperatures below 16 °C and affects both free-ranging and aquacultured fish [29]. Both CWD and RTFS significantly contribute to economic loss, with mortalities of fish affected by RTFS reaching up to 50% while CWD, a more chronic disease, results in 2–30% mortality in larger fish [28,30]. *F. psychrophilum* not only has ramifications in the aquaculture industry but also affects hatcheries used for stocking and rehabilitation purposes. To the authors’ knowledge, standard growth functions have yet to be applied to *F. psychrophilum*. The application of such functions can provide insight into the extent to which *F. psychrophilum* reacts to changes to its in vitro environment, including the effects of various antibiotics, through examination of the growth parameters in a given environment. This is especially relevant as antibiotic challenge tests are common practice in aquaculture, which is particularly important given the increasing resistance of *F. psychrophilum* to approved antibiotic drugs [31]. Given the limitations of standard functions modified to describe microbial growth and the challenges associated with the Baranyi equations, two novel models following an approach to derivation akin to that from which the Baranyi models were developed are presented and the resulting mathematical forms applied to data on *F. psychrophilum*.

## 2. Materials and Methods

### 2.1. Datasets

Eight datasets pertaining to *F. psychrophilum* grown on eight different liquid media, taken from two studies, were used for model evaluation. In Study 1, the first four datasets encompass growth of Strain SH3-81 of *F*. *psychrophilum* isolated from the kidney of juvenile coho salmon exhibiting classical bacterial cold-water disease (Sandy Hatchery, Oregon, USA). *F*. *psychrophilum* was plated on (1) tryptone yeast extract salts (TYES), (2) Shieh, (3) modified *Cytophaga* and (4) Cy7 broths [29,32,33,34]. The bacterium was inoculated in each of the four broth media and incubated for 24 h at 17 °C on a shaker prior to the start of the growth study. In duplicate, 1 mL of each broth culture was added to 50 mL of the corresponding broth medium using Nephelo culture flasks (Belco Glass, Inc., Vineland, NJ). Using a gyratory shaker (624 Environmental incubator shaker, New Brunswick Scientific Co., Inc., Edison, NJ), flasks were incubated at 17 °C and 1.136 g. Optical density was measured at 525 nm wavelength and recorded every 3–4 h for 72 h with datasets comprising 16 to 20 data points. See Holt [29] for further details concerning bacterial growth conditions. 

In Study 2, four datasets encompassing growth of *F*. *psychrophilum* strain Trouw 224 were isolated from diseased rainbow trout in Spain. Bacteria were pre-cultured in 25 mL of four liquid media, namely (5) tryptone–yeast extract–salts broth supplemented with glucose (FLPB), (6) TYES broth, (7) *Cytophaga* broth supplemented with carbohydrates and skimmed milk (CBCM), and (8) modified Anacker and Ordal broth (MAOB). These starting cultures were then inoculated (2 mL) into 200 mL of the respective liquid medium and incubated with 100 rpm shaking at 18 °C. Optical density was measured at 580 nm for 40 h with measurements being taken every 5 h. See Cepeda et al. [35] for further details concerning bacterial growth conditions.

The entirety of the observed growth values (Datasets 1–8) used in this study can be found in the Supplementary Materials Table S1. 

### 2.2. Mathematical Considerations

#### 2.2.1. Potential Growth

Following Baranyi et al. [23], we assume the potential rate of bacterial growth follows a logistic pattern. We formalize our assumption thus:

(1) $$\frac{dx}{dt}=\mu^{\prime }x,\;0\leq t<\infty$$

(2) $$\mu^{\prime }=\mu \left(1-\frac{x}{x_{m}}\right),\;0<x\leq x_{m},\;x\left(0\right)=x_{0},\;\mu >0$$

where *x* denotes microbial biomass at time *t*, $x_{0}$ is microbial biomass at time zero, $x_{m}$ is maximal microbial biomass as t→∞ , μ is a constant representing relative growth rate and $\mu^{\prime }$ relates rate of growth (dx/dt) to a specific microbial biomass (x). Substituting for *μ’* in Equation (1) using (2) and integrating analytically by the method of partial fractions gives *f*(*t*), the potential growth logistic function:

(3) $$x=\frac{x_{0}x_{m}}{x_{0}+\left(x_{m}-x_{0}\right)e^{-\mu t}}=f\left(t\right)$$

This equation can be re-parameterized as follows:

(4) $$x=\frac{p_{1}}{1+p_{2}e^{-p_{3}t}}$$

(5) $$p_{1}=x_{m},\;p_{2}=\frac{x_{m}}{x_{0}}-1,\;\;p_{3}=\mu$$

Substituting for *μ’* in Equation (1) and differentiating yields:

(6) $$\frac{1}{\mu }\frac{d^{2}x}{dt^{2}}=\left(1-\frac{2x}{x_{m}}\right)\frac{dx}{dt}$$

Equating Equation (6) to zero gives the point of inflexion (t*, x*) for *f*(*t*) at:

(7) $$x=x^{*}=\frac{x_{m}}{2}$$

This occurs when:

(8) $$t=t^{*}=\frac{1}{\mu }\ln\left(\frac{x_{m}-x_{0}}{x_{0}}\right)$$

#### 2.2.2. Actual Growth

We determine the actual rate of bacterial growth by dampening the potential rate, i.e.,

(9) $$\frac{dx}{dt}=\alpha \mu^{\prime }x,0\leq t<\infty$$

(10) $$\mu^{\prime }=\mu \left(1-\frac{x}{x_{m}}\right),0<x\leq x_{m},x\left(0\right)=x_{0},\mu >0$$

(11) α≡α(t),0<α≤1,α(0)=1,  α(∞)→0

The dampening factor *α*, which gives the instantaneous ratio of actual to potential growth rate, proposed herein is a monotonically decreasing function of time that mimics, for example, the effect of a build-up of an inhibitor on growth rate.

The lag phase may be identified as follows. The slope (*s*) of the actual growth curve *g*(*t*) at its point of inflexion (t*, x*) is obtained from Equations (9) to (11) as the product:

(12) $$s=\alpha \left(t^{*}\right)\times \mu^{\prime }\left(x^{*}\right)\times x^{*}$$

(13) $$s/x^{*}=\alpha \left(t^{*}\right)\times \mu^{\prime }\left(x^{*}\right)$$

Note that this product (Equation (12)) represents maximum growth rate. The equation of the tangent line, *y*(*t*), touching the steepest part of the curve (i.e., at the point of inflexion) is therefore:

(14) $$y-x^{*}=s\left(t-t^{*}\right)$$

The length of the lag phase, *T*, is determined by the point of intersection between this tangent line and the abscissa *x* = *x*_0_, with Equation (14) yielding:

(15) $$T=t^{*}-\frac{\left(x^{*}-x_{0}\right)}{s}$$

##### Rectangular Hyperbola

The dampening effect *α* can be represented by a rectangular hyperbola:

(16) $$\alpha =\frac{\lambda }{\lambda +t},\lambda >0$$

where *λ* is the point whereby the dampening effect is half maximal. Equation (16) is akin to the expression from enzyme kinetics for relative velocity in the presence of a noncompetitive inhibitor (see Segel [36]), assuming the instantaneous inhibitor concentration is directly proportional to time, and substituting for *t* in Equation (16). The actual growth function *g*(*t*) is obtained by substituting for *μ’* and *α* in Equation (9) using (10) and (16), then integrating analytically using partial fractions to give:

(17) $$x=\frac{x_{0}x_{m}}{x_{0}+\left(x_{m}-x_{0}\right){\left(\frac{\lambda }{\lambda +t}\right)}^{\lambda \mu }}\equiv g\left(t\right)$$

Equation (17) can be expressed in the re-parameterized form:

(18) $$x=\frac{p_{1}}{1+p_{2}{\left(1+p_{3}t\right)}^{-p_{4}}}$$

(19) $$p_{1}=x_{m},\;p_{2}=\frac{x_{m}}{x_{0}}-1,\;p_{3}=\frac{1}{\lambda },\;\;p_{4}=\lambda \mu$$

Differentiating Equation (9) having substituted for *μ’* and *α* yields:

(20) $$\frac{d^{2}x}{dt^{2}}=\mu \left(\frac{\lambda }{\lambda +t}\right)\left(1-\frac{1}{\lambda \mu }-\frac{2x}{x_{m}}\right)\frac{dx}{dt}$$

Therefore, a point of inflexion would exist at:

(21) $$x=x^{*}=\left(1-\frac{1}{\lambda \mu }\right)\frac{x_{m}}{2}$$

This would occur when:

(22) $$t=t^{*}=\lambda \left\{{\left[\frac{\left(\lambda \mu -1\right)\left(x_{m}-x_{0}\right)}{\left(\lambda \mu +1\right)x_{0}}\right]}^{1/\left(\lambda \mu \right)}-1\right\}$$

##### Simple Exponential

The dampening effect can also be represented by a simple exponential function:

(23) $$\alpha =e^{-\lambda t},\lambda >0$$

where *λ* is a rate parameter. Equation (23) states the ratio of actual to potential growth rate declines in a negative exponential manner over the growth period, possibly reflecting the deleterious build-up of some inhibitor. Substituting for *μ’* and *α* in Equation (9) using (10) and (23), then integrating analytically using partial fractions gives *g*(*t*):

(24) $$x=\frac{x_{0}x_{m}}{x_{0}+\left(x_{m}-x_{0}\right)\exp\left[-\frac{\mu }{\lambda }\left(1-e^{-\lambda t}\right)\right]}\;\equiv g\left(t\right)$$

Re-parameterizing Equation (24) gives:

(25) $$x=\frac{p_{1}}{1+p_{2}\exp\left[-p_{3}\left(1-e^{-p_{4}t}\right)\right]}$$

(26) $$p_{1}=x_{m},\;\;p_{2}=\frac{x_{m}}{x_{0}}-1,\;\;\;p_{3}=\frac{\mu }{\lambda },\;\;p_{4}=\lambda \;\;$$

Differentiating Equation (9) having substituted for *μ’* and *α* yields:

(27) $$\frac{d^{2}x}{dt^{2}}=\left[\mu e^{-\lambda t}\left(1-\frac{2x}{x_{m}}\right)-\lambda \right]\frac{dx}{dt}=0$$

Therefore, the point of inflexion (t*, x*) satisfies the pair of equations:

(28) $$x^{*}=\frac{x_{m}}{2}\left(1-\frac{\lambda }{\mu }e^{\lambda t^{*}}\right)$$

(29) $$t^{*}=-\frac{1}{\lambda }\ln\left\{1+\frac{\lambda }{\mu }\ln\left[\frac{x_{0}\left(x_{m}-x^{*}\right)}{x^{*}\left(x_{m}-x_{0}\right)}\right]\right\}$$

This pair of equations has to be solved using numerical methods [37].

The newly derived functions, herein referred to as logistic × hyperbola (log × hyp) and logistic × exponential (log × exp), were investigated along with popular growth functions (Table 1). The simple logistic was modified according to Zwietering et al. [12], where *x_m_* and *T* are as described above. In the Baranyi model, parameter *µ*_max_ is defined as the theoretical maximum relative rate, with *µ*_max_ also representing maximum relative rate in the modified logistic.

### 2.3. Model Fitting

For each of the eight datasets, microbial growth data were expressed on an absolute (untransformed) basis (optical density, OD) in addition to being expressed on a logarithmic basis [ln(OD/OD_0_)]. The BAR and MLOG models were derived and modified, respectively, to describe logarithmic transformed microbial growth data and therefore these models were fitted to the logarithmic transformed data. In contrast, the LOG, log × hyp and log × exp models were derived to describe absolute growth data and thus fitted to the untransformed data. All models were fitted by means of nonlinear regression using the Newton algorithm in the NLIN procedure of SAS [38]. A range of starting values for each parameter was determined through visual inspection of the growth curve. For parameter values that were difficult to estimate using visual inspection, *x_m_* and *x*_0_ were fixed in the first attempt at model fitting, providing an estimated starting value for the remaining parameters for future iterations whereby all parameters of the model were estimated. Using the range of starting values, PROC NLIN forms a grid and evaluates the model at each point on the grid. The values on the grid that yield the smallest objective function are used as initial parameter estimates for the first iteration of the fitting processes [38]. The range of starting values was the same between models that contained common parameters.

### 2.4. Statistical Analysis

Models were evaluated for goodness-of-fit along with analysis of their residuals using a range of statistical tests. Mean square prediction error (MSPE) is a measure of the expected mean squared difference between predicted and observed values. MSPE was calculated as the sum of the squared difference between predicted and observed values divided by the number of observations [39]. Agreement between model predictions and observed values was further evaluated using the concordance correlation coefficient (CCC), a single statistic which contains both accuracy and precision indicators [40]. CCC values were calculated according to Lin [40] with values of 1 representing perfect agreement, −1 perfect disagreement, and 0 no agreement between predicted and observed values [40]. Models’ goodness-of-fit for individual datasets were compared to one another using the Akaike information criterion (AIC). AIC is a test for model selection which accounts for goodness-of-fit while penalizing for over-fitting [41]. AIC was calculated for each model for a given dataset according to Akaike [41] with the model resulting in the smallest AIC being considered the best [41]. The accuracy factor (AF) averages the minimum ‘distance’ between each predicted data point and the line of equivalence. Thus, the accuracy factor is a measure of the average deviation of a model’s predictions and is used as a simple measure of the level of confidence in these predictions [42]. AF was calculated according to Baranyi et al. [43]. Calculated AF will always be greater than or equal to one, with larger values resulting from poorer fits.

The ability of each model to predict growth without systematically over- or under-estimating microbial mass was determined using the runs and Durbin–Watson (DW) tests. The number of runs test examines a sequence of residuals for unusual groupings of positive or negative residuals and tests against the null hypothesis that arrangement of signs is random. A run is a sequence of residuals with the same sign, positive or negative. Using the runs test, the probability of too few runs (indicating clustering of residuals with the same sign leading to systematic bias) or too many runs (indicating negative serial correlation) was determined [44]. Unlike the runs test, which ignores the actual size of the residual, the DW test examines dependencies in the error terms by testing for correlations between a residual and the residuals immediately before and after it in the sequence. The DW statistic (*d*), and upper (*d_u_*) and lower (*d_l_*) critical values were calculated according to Draper and Smith [44]. When *d* is less than the lower critical value *d_l_*, evidence of positive autocorrelation occurs, when the *d* value is greater than the upper critical value *d_u_*, evidence of negative autocorrelation occurs. The test is inconclusive when *d* falls between the upper and lower critical values. Positive serial autocorrelation in the residuals suggests the model has the tendency to systemically over- or under-estimate projected values [44]. The quantitative bias factor (BF) was calculated according to Ross [42]. Perfect agreement between predictions and observations will result in a BF of one. Values of BF greater than one occur when a model’s predictions are on average greater than observed values, while a value of less than unity occurs when a models’ predictions are on average less than observed values. When calculating the AF and BF statistics, both observed and predicted values underwent a ’*x* + 1’ transformation. This transformation allowed for the inclusion of data points whereby *x* = 0 when calculating these statistics. Likewise, data points that approached zero, and due to the logarithmic nature of the calculation result in nonsensical AF and BF values, were able to be included in the calculations following ‘*x* + 1’ transformation. 

### 2.5. Model Validation

As a means of cross-validation of proposed models, the statistic predicted residual error sum of squares (PRESS) was calculated. PRESS is based upon the leave-one-out or jackknife technique which evaluates the fit of a model to a sample of observations which in their entirety were not used for model development [45,46,47]. The basis of the statistic is the summing of the squared residuals resulting from systematically omitting individual observations, one by one, from a given dataset, while simultaneously refitting the proposed model to these reduced datasets. For example, if a dataset contained *n* data points, *n* datasets would be created from this one dataset, all differing by exactly one data point, with the model being fitted to these *n* datasets. After each individual fitting, the value of the *y*-variable is predicted at the omitted data point. The PRESS statistic was calculated according to Allen [45] providing an unbiased estimate of the model’s future prediction performance [47]. When fitting multiple models to the same dataset, the lowest PRESS value indicates the superior model. Additionally, CCC was used as a measure of goodness-of-fit between model predictions when fitted to datasets encompassing omitted data points and the corresponding observed values. 

## 3. Results

The suitability of the two new models derived (viz. log × hyp and log × exp) was assessed through their ability to mimic the growth of *Flavobacterium psychrophilum* cultivated on eight liquid media based upon absolute OD growth data. In addition, the simple logistic was fitted to these OD data, and its modified form plus the four-parameter Baranyi were fitted to the logarithmic transformation [ln(OD/OD_0_)] of the datasets as a means of affirmation. All five models examined in this study were evaluated based upon fitting behavior, examination of residuals, measures of goodness-of-fit and cross-validation.

### 3.1. Paramater Estimates

In general, all five growth functions fitted the data without major problems. Through visual inspection of all datasets, a range of initial parameter values was obtained for use with the grid search technique in the statistical software SAS. The resulting final values of lag time (*T*), maximum growth rate (*s*) and scaled maximum growth rate (*s*/x*) for the two new derivations in addition to the simple logistic (along with *µ*_max_ and *T* for BAR and MLOG) are presented in Table 2. Unlike MLOG and BAR, lag times for log × hyp and log × exp are not explicitly represented as a parameter in the equation and must be calculated using Equation (15). Likewise, maximum growth rate for log × hyp and log × exp is not explicitly represented as an equation parameter and therefore was calculated using Equation (12), while scaled maximum growth rate was calculated by dividing maximum growth rate by microbial biomass at the point of inflexion (Equation (13)). Estimates of *s, s*/x* and *T* were influenced by dataset. In Study 1, using OD data, the LOG, log × hyp and log × exp estimates of *s*, *s*/x* and *T* were in close agreement with one another. These three models determined the longest lag time and slowest maximum growth rate to occur when *F. psychrophilum* was cultured on the Cy7 medium (Table 2—Dataset 4). All three models were in agreement that fastest maximum growth rate and highest scaled maximum growth rate occurred when grown on the modified *Cytophaga* medium (Table 2—Dataset 3), with the shortest lag time and lowest scaled maximum growth occurring with the Shieh medium (Table 2—Dataset 2). When applied to logarithmic transformed OD data in Study 1, close agreement was found between the *µ*_max_ and *T* of the BAR and MLOG. In agreement with the log × hyp, log × exp and LOG, the models BAR and MLOG determined the longest lag time when *F. psychrophilum* was cultured on Cy7 (Table 2—Dataset 4). Both BAR and MLOG were in agreement that the shortest lag time occurred with the TYES medium (Table 2—Dataset 1). Highest relative growth rate with BAR and MLOG occurred on Shieh (Table 2—Dataset 2), with lowest relative growth rate occurring on Cy7 (Table 2—Dataset 4). It is important to note that *µ*_max_ of the Baranyi represents a maximum relative rate (units of per unit time, h^−1^), whereas the *s* of log × hyp and log × exp is a maximum rate (units of microbial biomass or its surrogate per unit time) and *s*/x* is a scaled maximum rate (units of per time, h^−1^).

In Study 2, LOG, log × hyp and log × exp were in agreement that the lowest maximum growth rate and shortest lag time occurred with the MAOB medium (Table 2—Dataset 8), with both log × hyp and log × exp determining lowest scaled maximum growth rate to occur on this medium. These three models determined maximum growth rate (absolute or scaled) was highest on the TYESB medium (Table 2—Dataset 6) and lag time longest on the CBCM medium (Table 2—Dataset 7). With both MLOG and BAR maximum relative growth rate occurred on CBCM (Table 2—Dataset 7), though the two values (0.847 and 1.66, respectively) were significantly different from one another. Also, with both models the lowest maximum relative growth rate occurred on the FLPB medium (Table 2—Dataset 5). Both models were in agreement that longest lag time occurred on MAOB (Table 2—Dataset 8) and shortest on FLPB (Table 2—Dataset 5).

Refer to Tables S2 and S3 in the Supplementary Materials for all of parameter estimates generated using log × hyp and log × exp when fitted to the eight liquid media datasets.

### 3.2. Growth Prediction

**Figure 1:**
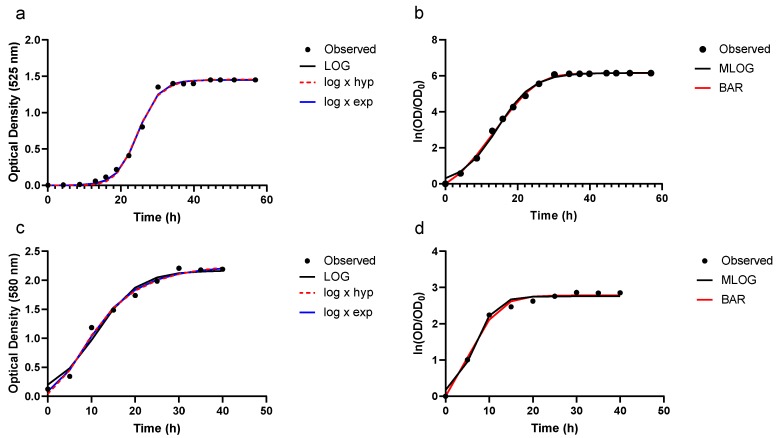
Optical density growth data and model predictions for *Flavobacterium psychrophilum*. Growth predictions using (**a**) LOG, log × hyp, log × exp and (**b**) MLOG and BAR, grown on a TYES liquid medium, and (**c**) LOG, log × hyp, log × exp and (**d**) MLOG and BAR, grown on a FLBP liquid medium. In panels (**a**) and (**c**), models are fitted to untransformed optical density measurements (OD) while (**b**) and (**d**) models are fitted to [ln(OD/OD_0_)].

Following parameter estimation, growth of *F. psychrophilum* was predicted as a function of time using the five growth equations, viz. Equations (3), (17) and (24) and those for MLOG and BAR displayed in Table 1. An example of observed and predicted absorbance values given by each growth function is presented in Figure 1. 

**Figure 2:**
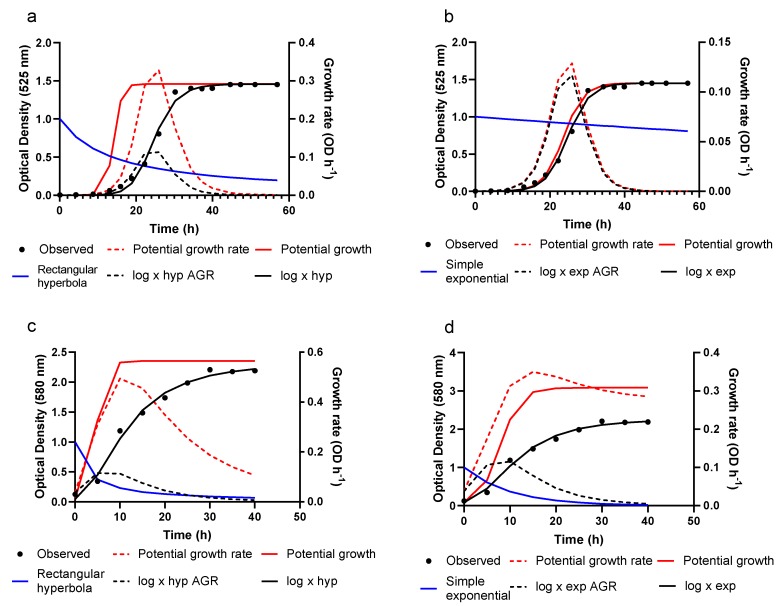
The effect of dampening potential growth rate, resulting actual growth, and actual rate of growth (AGR) of *Flavobacterium psychrophilum.* Dampening effect is represented by: a rectangular hyperbola (**a**), a simple exponential (**b**) when grown on a TYES liquid medium and a rectangular hyperbola (**c**), a simple exponential (**d**) when grown on FLBP liquid medium.

As seen in this figure, all five models appear to describe the observed growth data well with apparently little distinction between models. This figure also illustrates the effect that a logarithmic transformation has on the shape of the growth curve. The dampening effect of the log × hyp and log × exp functions, along with predicted growth, is shown in Figure 2.

**Figure 3:**
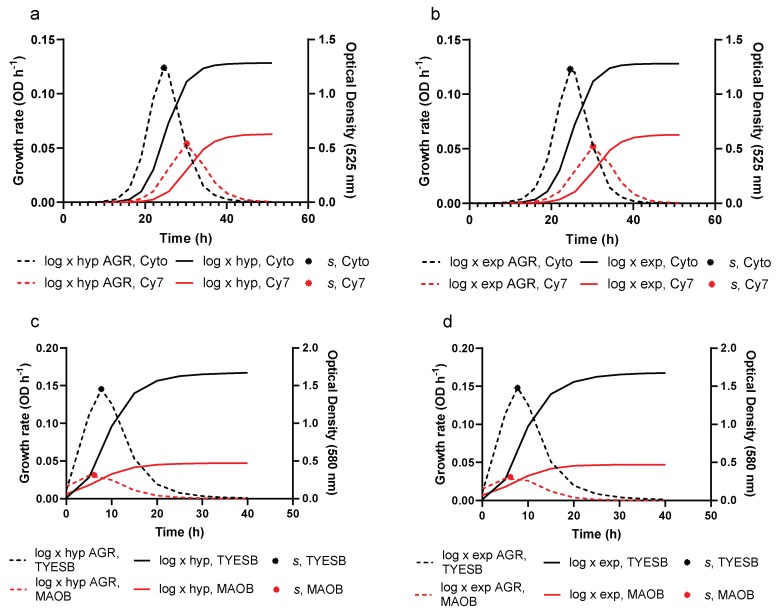
Comparison of actual growth rate (AGR), predicted growth and maximum actual growth rate (*s*) from liquid media resulting in highest and lowest maximum growth rates (*s*) from Study 1 and Study 2 when fitting log × hyp and log × exp. Panels (**a**) and (**b**) are taken from Study 1, and panels (**c**) and (**d**) from Study 2.

In this figure, potential growth rate and potential growth, represented by Equations (1) and (3) respectively, are presented for log × hyp and log × exp. Using either a rectangular hyperbola (Equation (16)) or a simple exponential (Equation (23)), potential growth rate is dampened resulting in actual growth rate (Equation (9)) for log × hyp and log × exp. Integrating Equation (9), the actual rate of growth, with the associated dampening functions (Equations (16) and (23)) results in Equations (17) and (24), representing OD as a function of time for log × hyp and log × exp, respectively. Refer to Figures S1–S6 in the Supplementary Materials for the figures corresponding to Figure 1 for the remaining liquid media. Comparison of growth and associated growth rates of *F. psychrophilum* between the two liquid media that resulted in the highest and lowest maximum growth rates (*s*) in Studies 1 and 2 obtained using Equation (12) for log × hyp and log × exp is presented in Figure 3. The interaction between growth rate and resulting growth can clearly be observed, in addition to the time at which *s* occurs coinciding with the point of inflexion of the respective growth curve.

### 3.3. Model Evaluation

Models were evaluated for goodness-of-fit using four criteria: Akaike information criterion, mean square prediction error, concordance correlation coefficient and accuracy factor. The goodness-of-fit values for the five models were compared over studies in addition to being averaged over the eight datasets.

The averaged AIC, MSPE, CCC and AF values resulting from fitting the five models to the four datasets of Study 1 and the four datasets of Study 2, in addition to the totaled average over the eight datasets, are given in Table 3. The BAR resulted in a smaller averaged AIC (−55.7 ± 5.6) value in comparison with MLOG (−51.6 ± 3.7), when comparing across all eight datasets. In Study 1, BAR (−67.5 ± 5.5) resulted in a smaller averaged AIC value compared with MLOG (−58.8 ± 3.1), while very similar AIC values were observed between BAR (−44.0 ± 2.9) and MLOG (−44.4 ± 3.6) in Study 2. Comparing AIC values resulting from models fitted to the original, rather than the logarithmic transformed data, viz. LOG, log × hyp and log × exp, the LOG (–80.8 ± 15.1) gave the smallest AIC value averaged across all eight datasets, followed by log × exp (–78.3 ± 14.0) and log × hyp (−77.0 ± 14.0). The LOG (−117.1 ± 11.5) resulted in the lowest averaged AIC value in Study 1, followed by log × exp (−111.6 ± 11.3) and log × hyp (−110.0 ± 11.5). In contrast, log × exp (−45.0 ± 2.9) resulted in the lowest AIC value in Study 2 followed by LOG (−44.6 ± 3.9) and log × hyp (–43.9 ± 2.7). Based upon MSPE, the BAR (0.009 ± 0.003) outperformed MLOG (0.013 ± 0.004) in both studies. LOG (0.001 ± 0.0002), log × hyp (0.001 ± 0.0003) and log × exp (0.001 ± 0.0003) performed equally well in Study 1, while in Study 2 both log × exp (0.003 ± 0.001) and log × hyp (0.004 ± 0.001) resulted in lower averaged MSPE compared with LOG (0.005 ± 0.002). Both log × exp (0.002 ± 0.001) and log × hyp (0.002 ± 0.001) resulted in lower averaged MSPE values compared to LOG (0.003 ± 0.001) when averaged over all eight datasets. 

Similar to the results found for MSPE, the LOG, log × hyp and log × exp performed equally well (0.998 ± 0.001) on the basis of CCC when fitting the datasets of Study 1. When fitting the datasets of Study 2, both log × hyp (0.988 ± 0.005) and log × exp (0.989 ± 0.005) gave higher CCC values compared to LOG (0.987 ± 0.004), with higher averaged CCC values for both log × hyp (0.993 ± 0.003) and log × exp (0.993 ± 0.003) compared to LOG (0.992 ± 0.003) over the eight datasets. The BAR resulted in higher CCC values in both Study 1 and 2 compared to MLOG with averaged values of 0.998 ± 0.001 and 0.997 ± 0.001, respectively. On the basis of AF, BAR (1.024 ± 0.005) performed better than MLOG (1.039 ± 0.005) in Study 1 in addition to Study 2, BAR (1.016 ± 0.003) MLOG (1.022 ± 0.007). When averaged over the eight studies, BAR (1.020 ± 0.003) resulted in a lower AF compared to MLOG (1.031 ± 0.005). In Study 1, LOG (1.013 ± 0.002) performed slightly better than log × exp (1.015 ± 0.002) and log × hyp (1.016 ± 0.003) on the basis of AF index. In Study 2, log × exp (1.027 ± 0.002) resulted in the smallest AF, followed by log × hyp (1.030 ± 0.003) and LOG (1.033 ± 0.005). When averaged over all eight datasets, log × exp (1.021 ± 0.002) resulted in the smallest AF statistic, followed by log × hyp (1.023 ± 0.003) and LOG (1.023 ± 0.005).

Residuals were examined through clustering of same signed residuals and through serial correlation, with results of the runs test and the DW test for serial correlation presented in Table 4. Distribution of runs of signs was examined according to Draper and Smith [44]. None of the five models showed a significant number of too many runs of signs (data not shown). Of the eight datasets, runs of signs were deemed to be random in all eight datasets for both BAR and MLOG. In contrast, too few runs were observed with LOG, log × hyp and log × exp in 2, 3 and 2 of the datasets, respectively. Runs of signs were found to be random with the remaining datasets. Serial correlation was investigated using the DW statistic and the number of times a specific model gave positive or no serial correlation when fitted to a given dataset is shown in Table 4. Both MLOG and BAR displayed no serial correlation when fitted. The remaining three models showed no serial correlation for seven of the eight datasets, with these models exhibiting positive serial correlation for one of these datasets. Interestingly, evidence of serial correlation in the residuals for LOG, log × hyp and log × exp all occurred with the same dataset, viz. Dataset 4. Comparing MLOG to BAR on the basis of BF index, the two models had a tendency to overestimate observations in Study 1 and in Study 2, with BAR resulting in a BF closer to 1 in both studies. In Study 1, LOG, log × hyp and log × exp had a tendency to slightly underestimate observed values with all three models resulting in BF below 1, 0.996 ± 0.002, 0.993 ± 0.001 and 0.994 ± 0.000, respectively. In Study 2, LOG slightly overestimated observed values (1.001 ± 0.003) while both log × hyp (0.994 ± 0.002) and log × exp (0.994 ± 0.003) underestimated them. When averaged over all eight datasets, LOG resulted in a value just below 1, (0.999 ± 0.002) while log × hyp (0.994 ± 0.001) and log × exp (0.991 ± 0.001) also slightly underestimated observed values on average.

### 3.4. Model Validation

Using the leave-one-out/jackknife technique, the models were successively fitted to sub-datasets of Dataset 1 and 2 from Study 1 and Dataset 5 and 6 from Study 2. PRESS and CCC statistics were calculated from model predictions and observed values for these sub-datasets (Table 5). When examining the PRESS statistic, the lowest value indicates the best future prediction performance while CCC presents a clear indication of prediction precision and accuracy. The log × exp resulted in the lowest PRESS value in three of the four datasets with LOG resulting in the lowest value in the other. Examining CCC, log × exp resulted in the best CCC value in three of the four datasets with LOG giving the highest in the other. Both log × exp (0.9814, 0.9808) followed by log × hyp (0.9782, 0.9807) resulted in higher CCC values when fitted to the sub-datasets from Dataset 5 and 6 compared to LOG (0.9726, 0.9806).

## 4. Discussion

Simple mechanistic growth models are derived from rate:state principles represented as a differential equation relating growth rate to organism or population size. Equations expressed in this form allow straightforward biological interpretation of the parameters, and in many cases, can be solved analytically. Following this methodology, classical growth equations have been derived including the logistic, Gompertz, and Richards [20,21,48]. In an effort to apply them to bacterial growth curves, Zwietering et al. [12] modified these functions in such a way as to ascribe increased biological meaning to some of the parameters, resulting in the modified logistic, Gompertz and Richards. The modified logistic has been shown to describe microbial growth with more reliable kinetic parameters estimates compared to the modified Gompertz [6,49]. As such the modified logistic, viz. MLOG, was chosen as a basis for model comparison along with BAR in this study.

The Baranyi family of equations [2,15,23], in addition to the model of Huang [18,19], represent two of very few attempts to derive growth functions mechanistically to describe bacterial growth. The Baranyi models are based upon the concept of potential and actual growth, with actual growth being less than potential as the bacteria adapt from the pre- to post-inoculation environment. Potential growth is represented by the first-order differential equation dx/dt=μ(x)x whereby *x* denotes cell concentration and *µ*(*x*) denotes relative growth rate [23]. The differential equation adopted in the Baranyi models is akin to the logistic. However, due to food microbiologists’ preference for using the logarithm of cell concentration, the logarithmic transformation of microbial cell concentration was related to incubation time [2]. This transformation departs from the mechanistic derivation of the logistic and hinders the model’s application to absolute, non-transformed growth data. As actual growth is less than potential, an adjustment function is incorporated into the differential equation representing growth potential. The resulting differential equation for actual bacterial growth therefore takes the form $dx/dt=\mu_{\max}\alpha \left(t\right)u\left(x\right)x$ with α(t) representing the adjustment function and u(x) the so-called inhibition function. The authors direct readers to Baranyi et al. [23] and Baranyi and Roberts [2] for derivation of this function and all other mathematical considerations. The differential equation representing actual growth can be solved analytically resulting in a six-parameter equation [2]. However, due to fitting difficulties associated with the large number of parameters, and sensitivity to number of data points, certain assumptions were implemented to reduce the original Baranyi to a four-parameter equation [2,24,25]. The resulting four-parameter model BAR is the most widely applied of the Baranyi family of models and one of the models investigated herein [7]. Like the Baranyi family of models, the Huang model is also mechanistically derived to describe bacterial growth. While the Baranyi takes into account the pre- and post-inoculation state of the cells on growth, Huang [18] aimed at developing a model that described bacterial growth on the basis of current growth environment. However, the discontinuous nature of the model in describing the three phases of growth, viz. lag, exponential and stationary, requires the use of a transitional function. In addition to complicating the mathematics in manipulating the analytical solution to the growth function, the transitional function introduces a non-biological parameter into the model.

Two new models, based upon the principles promulgated by Baranyi and co-workers, aimed at describing bacterial growth are presented in this paper. These growth functions, making use of the concepts of potential and actual growth, utilize a mathematical function to dampen potential logistic growth. Dampening effects are represented by either a rectangular hyperbola or a simple exponential. These dampening functions are incorporated into the basic differential equation which is then solved analytically resulting in two growth equations (viz. log × hyp and log × exp). The use of these seemingly empirical dampening functions results in growth equations that can be expressed as relatively simple analytical expressions. The Baranyi makes use of an adjustment function to take into account the effects of pre- and post-inoculation environments on growth. Like the log × hyp and log × exp models, potential growth in the Baranyi is represented by a first-order differential equation. Actual growth is given by incorporating an adjustment function into this first-order differential equation. The adjustment function of Baranyi and Roberts [2] is based upon the principle that growth during the lag phase is inhibited by a ‘bottle-neck’ intracellular substance following Michaelis-Menten kinetics [2]. The adjustment function takes the form: $\alpha \left(t\right)=\frac{q_{0}}{q_{0}+e^{-vt}}$, a two-parameter function with a rate parameter (*v*) and a constant ($q_{0}$) representing the physiological state of the inoculum at time zero. However, as the adjustment function gives no indication of what the critical factor causing the ‘bottle-neck’ could be, the adjustment function of the Baranyi, like dampening functions of log × hyp and log × exp, is essentially a quasi-mechanistic expression with biological meaning being prescribed to it [24]. In contrast to the two novel models proposed (viz. log × hyp and log × exp), the adjustment function and ordinary differential equation of the Baranyi are integrated separately resulting in *A*(*T*), the explicit solution to the adjustment function, and *y*(*t*) the explicit solution to the ordinary differential equation and are derived specifically to describe logarithmic transformed microbial concentration data. These two equations result in the six-parameter model of Baranyi and Roberts [2]. Although more biologically thorough, the six-parameter Baranyi model is generally difficult to fit, leading to the previously mentioned re-parameterization and subsequent fixing of two shape parameters, resulting in the more widely used and robust four-parameter Baranyi model BAR. See Table 1 for explicit solutions to BAR. Regardless, the solution to BAR is still quite convoluted in comparison to the explicit solutions to log × hyp and log × exp, complicating the mathematics involved when manipulating this equation.

In contrast to BAR and MLOG, *T* is not explicitly represented in our LOG, log × hyp and log × exp models. Instead it has to be calculated as the intercept between the tangent to the steepest part of the growth equation and initial microbial biomass. When grown on the TYES liquid medium (Dataset 1), LOG, log × hyp and log × exp determined lag times to be 18.8, 18.7 and 18.3 h respectively, with all three models in agreement with maximum growth rate (units of microbial biomass per h) of 0.122 achieved on this medium. In terms of scaled maximum growth rate (h^−1^), LOG, log × hyp and log × exp were in close agreement with values of 0.17, 0.18 and 0.17 when grown on the TYES liquid medium. In comparison, when MLOG and BAR were fitted to the logarithmic transformation of this same dataset, lag times were determined to be 4.6 and 3.7 h, respectively. Maximum relative growth rates (h^−1^) of MLOG and BAR were determined to be 0.314 and 0.292, respectively. Due to the logarithmic transformation effecting the overall shape of a growth curve, it is clear that lag times are not comparable between models applied to absolute OD data and logarithmic transformed data, viz. LOG, log × hyp and log × exp versus MLOG and BAR, respectively. However, BAR and MLOG estimate lag times that are very much shorter than lag times when using the absolute untransformed OD data. Lag time estimates of MLOG and BAR are much shorter than the time at which OD starts to rise over the baseline OD_0_ using the actual untransformed data; therefore these two models appear to underestimate the real lag time. This can be seen through comparison of lag time values in Table 2 and visualization of Figure 1, in addition to the corresponding figures (Figures S1–S6 in the Supplementary Materials). The extent of underestimation of lag time for BAR and MLOG is more apparent in Study 1 compared to Study 2, whereby Study 1 is characterized by lower *x*_0_ values and a more prolonged lag phase.

The models MLOG and BAR represent the maximum growth parameter as a relative rate (h^−1^), while LOG, log × hyp and log × exp represent maximum growth in an absolute sense (units of microbial biomass per h) in addition to being able to represent maximum growth on a relative basis through scaled maximum growth rate (h^−1^). Very little information exists concerning parameters estimates resulting from models fitted to bacterial growth data in the field of aquaculture, with even less being available concerning *F. psychrophilum*. Stenholm et al. [50] reported maximum growth rates between 0.062 and 0.098 h^−1^ for 26 strains of *F. psychrophilum*. It is unclear however if these values were generated using a model, with the authors describing growth rates as being determined from the exponential increase in OD at 525 nm. Growth rates of two pathogenic bacteria *Aeromonas hydrophila* and *Vibrio alginolyticus*, obtained by fitting modified forms of the logistic and Gompertz, have been published [5]. However, the authors omitted the units when describing parameter estimates. Clearly, many factors must be standardized before meaningful comparisons of model-derived growth parameter estimates can be made. These factors include but are not limited to: method by which bacterial growth is measured, transformation applied to the dependent variable, and actual definition of growth rate [7].

The shortcomings of measuring microbial growth using the OD technique are worthy of note. The ability to derive lag and rate parameters from OD experiments is limited by the relatively high (~10^6^–10^7^ CFU/mL detection thresholds of OD measurement devices [4]. Therefore, this technique is best suited in circumstances whereby high cell densities are reached. In the field of predictive food microbiology spoilage resulting from bacterial load is a concern at high levels, making the absorbance technique practical in this field of study [4]. Likewise, high levels of the pathogenic bacteria used in this current study, *F. psychrophilum,* are commonly incubated to elicit typical clinical signs of rainbow trout fry syndrome. In a challenge test, Aoki et al. [51] inoculated ~10^6^ CFU/mL of *F*. *psychrophilum* on a modified *Cytophaga* liquid medium, the same media as in Dataset 3 of this study. Bacteria reached the exponential phase of growth, at CFU/mL of ~10^7^, and subsequently experimental fish were challenged with this load resulting in clinical signs of disease. Therefore, it is it feasible that the relatively high level of *F*. *psychrophilum* used in studies permits the use of the absorbance technique to determine lag and rate parameter estimates when studying this bacterium.

On the basis of goodness-of-fit and cross-validation, all the models fitted the data well. Compared to LOG and MLOG, the models log × hyp, log × exp and BAR are characterized by a flexible point of inflexion. In contrast, LOG and MLOG have fixed points of inflexion. For a logistic function, the fixed point of inflexion results in a symmetric sigmoidal curve. The major drawback of a fixed point of inflexion is that growth parameters are estimated at this maximum rate which always occurs at 0.5 of *x_m_* for the logistic [14]. The remaining equations display flexible points of inflexion that result in a more robust and versatile growth curve. The point of inflexion of these growth functions can all be calculated by equating the second derivative of the function to zero and solving for x* (the microbial biomass at the point of inflexion). However, log × hyp is the only equation out of log × hyp, log × exp and BAR whereby t* (time at which the point of inflexion occurs) and x* can be solved for analytically, with log × exp requiring numerical methods to solve for t* and the second derivative of the BAR unable to be solved in such a way that it is explicit in *t*, the independent variable [7]. Therefore, without the ability to solve for t* for the Baranyi, it is not possible to calculate maximum rate of actual growth using $dx/dt=\mu_{\max}\alpha \left(t^{*}\right)u\left(x^{*}\right)x^{*}.$ The ability of a growth function to be easily manipulated mathematically has important ramifications for their application and is an example of the advantage of log × hyp over the other functions examined.

The dampening functions of log × hyp and log × exp and the adjustment function of BAR both delay and suppress the exponential phase of growth. This is can be seen through visualization of Figure 2, where potential growth, represented by a logistic, is suppressed by a dampening function resulting in actual growth represented by log × hyp and log × exp. In addition, the resulting actual growth rate of log × hyp and log × exp is presented following suppression of the potential growth rate of the logistic. Using the parameter estimates from fitting log × hyp and log × exp to a given dataset, actual growth rate (Equation (9)) can be calculated and plotted as a function of time. The ability of log × hyp and log × exp to be manipulated mathematically allows for comparison of how growing conditions influence maximum rate of growth (*s*), absolute growth and rate of growth as displayed in Figure 3. The dampening functions of log × hyp and log × exp, and the adjustment function of BAR both suppress potential logistic growth but do so in different ways. The adjustment function of the Baranyi monotonically increases from a positive fraction to one, and represents the gradual adaptation of the bacteria to attain the maximum specific growth rate, as affected by their physiological state before and after inoculation. In contrast, the dampening functions of log × hyp and log × exp monotonically decrease from one to zero and represent the effect of culture conditions on growth rate. While the dampening functions of log × hyp and log × exp, viz. a rectangular hyperbola and a simple exponential, are seemingly empirical in nature some mechanistic meaning can be ascribed to them. For example, they may represent the effect of the build-up of an inhibitor on growth rate, or the effect of changes in growing conditions (e.g., acidity, temperature, water activity, oxygen levels) on growth rate over time. Indeed, a rectangular hyperbola is the standard representation in enzyme kinetics for relative velocity in the presence of a noncompetitive inhibitor [36]. The ability to describe these effects on bacterial growth rate add a level of novelty to this family of models. Without the adjustment function in BAR, and the dampening function of the log × models, these equations revert to a standard logistic form.

## 5. Conclusions

The objective of our research was to assess the ability of two novel functions, whose derivations are based upon principles promulgated by Baranyi and co-workers, to describe bacterial growth of the fish pathogen *F. psychrophilum*. Models were evaluated on the basis of statistical measures of goodness-of-fit, cross-validation and examination of residuals. Due to the nature of their derivation, log × hyp, log × exp and LOG were fitted to bacterial growth data in terms of measured OD over time, while BAR and MLOG were fitted to the logarithmic transformation of these data. Not only do log × hyp, log × exp and BAR perform better, or equally well, compared to their logistic counterparts (LOG and MLOG) in their ability to describe the time evolution in microbial mass, they also provide insight into the mechanisms that are driving bacterial growth through the concepts of potential and actual growth. The adjustment function of the Baranyi represents the suppression of growth that occurs when a bacterium adjusts from the pre- to post-inoculation environment, with the Baranyi model successfully describing bacterial growth under changing temperatures. Similarly, the two new models presented herein, log × hyp and log × exp, contain a dampening function, which represents the depression in the potential logistic growth, and theoretically encompasses a bacterium’s growth response to changing growing conditions. The two novel models have successfully described bacterial growth under constant external environmental conditions. The two elementary dampening functions represented in these equations, a rectangular hyperbola and a single exponential function, result in simple analytical equations in comparison with the existing Baranyi family of models in addition to describing rates of growth in both absolute and relative terms. From LOG and log × hyp functions it is possible to derive simple algebraic expressions to calculate inflexion point, maximum growth rate and scaled maximum growth rate. Based on fitting and predictive performance, the two newly derived models can be considered suitable alternatives to classical functions to describe bacterial growth and estimate key growth parameters.

## Figures and Tables

**Table 1:** Evaluated growth functions.

	Functional Form
Simple logistic (LOG)	$x=\frac{x_{0}x_{m}}{x_{0}+\left(x_{m}-x_{0}\right)e^{-\mu t}}$
Modified logistic (MLOG)	$x=\frac{x_{m}}{\left\{1+\exp\left[\frac{4\mu_{\max}}{x_{m}}\left(T-t\right)+2\right]\right\}}$
Baranyi four parameter (BAR)	$x=x_{0}+\mu_{\max}A\left(t\right)-\ln\left[1+\frac{e^{\mu_{\max}A\left(t\right)}-1}{e^{\left(x_{m}-x_{0}\right)}}\right]$ $A\left(t\right)=t+\frac{1}{\mu_{\max}}\ln\left[e^{-\mu_{\max}t}+e^{-h_{0}}-e^{-\mu_{\max}t-h_{0}}\right]$ where $h_{0}=$*µ*_max_*T =* ln[1 *+*$\frac{1}{q_{0}}]$
log × hyp	$x=\frac{x_{0}x_{m}}{x_{0}+\left(x_{m}-x_{0}\right){\left(\frac{\lambda }{\lambda +t}\right)}^{\lambda \mu }}$
log × exp	$x=\frac{x_{0}x_{m}}{x_{0}+\left(x_{m}-x_{0}\right)exp\left[-\frac{\mu }{\lambda }\left(1-e^{-\lambda t}\right)\right]}$

**Table 2:** Comparison of maximum growth rate (*s*, units of microbial mass per h), scaled maximum growth rate (*s*/x*, h^−1^), lag time (*T*, *h*) and maximum relative growth rate (*µ*_max_, h^−1^), generated by the standard logistic (LOG), logistic × hyperbola (log × hyp) and logistic × exponential (log × exp) fitted to optical density (OD) growth data and the modified logistic (MLOG) and four-parameter Baranyi (BAR) fitted to logarithmic transformed growth data [ln(OD/OD_0_)].

	LOG			log × hyp			log × exp			MLOG		BAR	
Dataset	*s*	s/x*	*T*	*s*	s/x*	*T*	*s*	s/x*	*T*	*µ*_max_	*T*	*µ*_max_	*T*
1	0.122	0.168	18.8	0.122	0.182	18.7	0.122	0.170	18.3	0.314	4.6	0.292	3.7
2	0.096	0.138	16.8	0.095	0.151	16.6	0.097	0.163	17.1	0.362	5.6	0.348	5.3
3	0.122	0.191	19.8	0.124	0.207	19.8	0.123	0.197	19.5	0.302	5.5	0.291	5.1
4	0.052	0.165	24.5	0.053	0.181	24.7	0.052	0.168	24.2	0.219	7.8	0.221	7.7
5	0.111	0.103	3.1	0.124	0.174	1.7	0.137	0.161	1.6	0.285	1.7	0.334	1.8
6	0.133	0.161	3.7	0.145	0.225	3.5	0.148	0.237	3.6	0.309	2.9	0.506	4.4
7	0.120	0.206	4.1	0.119	0.217	4.0	0.107	0.279	4.8	0.847	7.6	1.656	8.3
8	0.031	0.130	1.4	0.031	0.146	1.2	0.030	0.130	0.7	0.457	7.7	1.452	8.7

**Table 3:** Goodness-of- fit: Akaike information criterion (AIC), mean square prediction error (MSPE) concordance correlation coefficient (CCC) and accuracy factor (AF) values obtained when fitting models to microbial growth data from Study 1 and 2.

Measure of Goodness-of-Fit	LOG	log × hyp	log × exp	MLOG	BAR
AIC					
Study 1-Average (±SE)	−117.1 (11.5)	−110.0 (11.5)	−111.6 (11.3)	−58.8 (3.1)	−67.5 (5.5)
SStudy 2-Average (±SE)	−44.6 (3.9)	−43.9 (2.7)	−45.0 (2.9)	−44.4 (3.6)	−44.0 (2.9)
SOverall-Average (±SE)	−80.8 (15.1)	−77.0 (14.0)	−78.3 (14.0)	−51.6 (3.7)	−55.7 (5.6)
MSPE					
SStudy 1-Average (±SE)	0.001 (0.0002)	0.001 (0.0003)	0.001 (0.0002)	0.024 (0.003)	0.014 (0.004)
SStudy 2-Average (±SE)	0.005 (0.002)	0.004 (0.001)	0.003 (0.001)	0.005 (0.002)	0.004 (0.001)
SOverall-Average (±SE)	0.003 (0.001)	0.002 (0.001)	0.002 (0.001)	0.013 (0.004)	0.009 (0.003)
CCC					
SStudy 1-Average (±SE)	0.998 (0.001)	0.998 (0.001)	0.998 (0.001)	0.997 (0.001)	0.998 (0.001)
SStudy 2-Average (±SE)	0.987 (0.004)	0.988 (0.005)	0.989 (0.005)	0.997 (0.001)	0.998 (0.001)
SOverall-Average (±SE)	0.992 (0.003)	0.993 (0.003)	0.993 (0.003)	0.997 (0.001)	0.998 (0.001)
AF					
SStudy 1-Average (±SE)	1.013 (0.002)	1.016 (0.003)	1.015 (0.002)	1.039 (0.005)	1.024 (0.005)
SStudy 2-Average (±SE)	1.033 (0.005)	1.030 (0.003)	1.027 (0.002)	1.022 (0.007)	1.016 (0.003)
SOverall-Average (±SE)	1.023 (0.005)	1.023 (0.003)	1.021 (0.002)	1.031 (0.005)	1.020 (0.003)

**Table 4:** Examination of residuals using the runs test, Durbin–Watson (DW) and averaged bias factor (BF) statistics obtained when fitting models to growth data from Study 1 and 2.

Test for Examination of Residuals	LOG	log × hyp	log × exp	MLOG	BAR
Runs test					
Runs were random	6	5	6	8	8
Too few runs	2	3	2	0	0
No. curves exhibiting serial correlation determined by DW statistic (*α* = 0.01)					
No serial correlation	7	7	7	8	8
Positive Correlation	1	1	1	0	1
BF					
SStudy 1-Average (±SE)	0.996 (0.002)	0.993 (0.001)	0.994 (0.000)	1.017 (0.001)	1.003 (0.001)
SStudy 2-Average (±SE)	1.001 (0.003)	0.994 (0.002)	0.994 (0.003)	1.006 (0.003)	1.001 (0.000)
SOverall-Average (±SE)	0.999 (0.002)	0.994 (0.001)	0.994 (0.001)	1.011 (0.003)	1.002 (0.001)

**Table 5:** Predicted residual error sum of squares (PRESS) and concordance correlation coefficient (CCC) resulting from cross-validation of Datasets 1-2 from Study 1 and Datasets 5-6 from Study 2.

Cross-Validation Test	Model		
	LOG	log × hyp	log × exp
PRESS			
Dataset 1	0.0717	0.0718	0.0599 *
Dataset 2	0.0110 *	0.0125	0.0134
Dataset 5	0.2640	0.2135	0.1858 *
Dataset 6	0.1184	0.1153	0.1149 *
CCC			
Dataset 1	0.9945	0.9945	0.9953 *
Dataset 2	0.9990 *	0.9989	0.9988
Dataset 5	0.9726	0.9782	0.9814 *
Dataset 6	0.9806	0.9807	0.9808 *

(*) represents the model which performed best, on the basis of PRESS and CCC, for a given dataset.

## Supplementary Materials

The following are available online at https://www.mdpi.com/2076-2615/10/3/435/s1, **Table S1**: Observed growth data of *Flavobacterium psychrophilum* on eight liquid mediums, (1) TYES, (2) Shieh, (3) modified Cytophaga, (4) Cy7, (5) FLPB, (6) TYESB, (7) CBCM and (8) MAOB; **Table S2**: Resulting parameter estimates of log × hyp fit to *Flavobacterium psychrophilum* grown on eight liquid mediums: (1) TYES, (2) Shieh, (3) modified Cytophaga, (4) Cy7, (5) FLPB, (6) TYESB, (7) CBCM and (8) MAOB; **Table S3**: Resulting parameter estimates of log × exp fit to *Flavobacterium psychrophilum* grown on eight liquid mediums: (1) TYES, (2) Shieh, (3) modified Cytophaga, (4) Cy7, (5) FLPB, (6) TYESB, (7) CBCM and (8) MAOB; **Figure S1**: Optical density growth data and model predictions for *Flavobacterium psychrophilum*. Growth predictions using (a) LOG, log × hyp, log ×exp (b) MLOG and BAR grown on a Sheih liquid medium; **Figure S2:** Optical density growth data and model predictions for *Flavobacterium psychrophilum*. Growth predictions using (a) LOG, log × hyp, log × exp (b) MLOG and BAR grown on a modified Cytophaga liquid medium; **Figure S3**: Optical density growth data and model predictions for *Flavobacterium psychrophilum*. Growth predictions using (a) LOG, log × hyp, log × exp (b) MLOG and BAR grown on a Cy7 liquid medium; **Figure S4**: Optical density growth data and model predictions for *Flavobacterium psychrophilum*. Growth predictions using (a) LOG, log × hyp, log × exp (b) MLOG and BAR grown on a TYESB liquid medium; **Figure S5**: Optical density growth data and model predictions for *Flavobacterium psychrophilum*. Growth predictions using (a) LOG, log × hyp, log × exp (b) MLOG and BAR grown on a CBCM liquid medium; **Figure S6**: Optical density growth data and model predictions for *Flavobacterium psychrophilum*. Growth predictions using (a) LOG, log × hyp, log × exp (b) MLOG and BAR grown on a MAOB liquid medium.
