# Supplementary material for: New Insights into Modelling Bacterial Growth with Reference to the Fish Pathogen Flavobacterium psychrophilum
Source: Animals (Basel). 2020 Mar 5;10(3):435. doi: 10.3390/ani10030435 (PMC7143051; doi:10.3390/ani10030435)
Supplement: Supplementary file 1 [file animals-10-00435-s001.pdf]

Table S1. Observed growth data of *Flavobacterium psychrophilum* on eight liquid mediums, (1) TYES, (2) Shieh, (3) modified Cytophaga, (4) Cy7, (5) FLPB, (6) TYESB, (7) CBCM and (8) MAOB.

(1) TYES

Time	OD	$\ln(OD/OD_0)$
0	0.0031	0
4	0.0055	0.57335
9	0.0128	1.41804
13	0.0588	2.94274
16	0.1145	3.60917
19	0.2187	4.2563
22	0.41	4.88476
26	0.8048	5.55919
30	1.3523	6.07816
34	1.4007	6.11333
37	1.3983	6.11161
40	1.4007	6.11333
45	1.4516	6.14902
47	1.4516	6.14902
51	1.4516	6.14902
57	1.4516	6.14902

(2) Shieh

Time	OD	$\ln(OD/OD_0)$
0	0.0031	0
5	0.0055	0.57335
9	0.008	0.94804
13	0.0637	3.02278
16	0.1436	3.83563
19	0.2817	4.50944
22	0.4657	5.01214
26	0.8509	5.61489
30	1.1924	5.95232
34	1.3014	6.03979
37	1.3014	6.03979
40	1.3498	6.07631
44	1.3983	6.11161
47	1.3983	6.11161
51	1.3983	6.11161
57	1.3983	6.11161

(3) modified Cytophaga

Time	OD	$\ln(OD/OD_0)$
0	0.0031	0
4	0.0055	0.57335
9	0.0104	1.2104
13	0.037	2.47952
16	0.0709	3.12987
19	0.1654	3.97696
22	0.3083	4.59967
26	0.6523	5.3491
30	1.1997	5.95842
34	1.2554	6.00381
37	1.3014	6.03979
40	1.2554	6.00381
44	1.2529	6.00181
47	1.2529	6.00181
51	1.2529	6.00181

(4) Cy7

Time	OD	$\ln(OD/OD_0)$
0	0.0031	0.00001
4	0.0031	0.00006
9	0.008	0.00032
13	0.0104	0.0015
16	0.0249	0.00417
19	0.0419	0.01218
22	0.0588	0.0321
26	0.1121	0.10615
30	0.2696	0.29
34	0.4827	0.4856
37	0.5747	0.56139
40	0.6256	0.60137
44	0.6426	0.61953
47	0.6571	0.62447
51	0.6426	0.62682
57	0.628	0.62756
60	0.6183	0.62766
64	0.5965	0.6277
67	0.599	0.62771
70	0.599	0.62772

(5) FLPB

Time	OD	$\ln(OD/OD_0)$
0	0.126	0
5	0.344	1.00436
10	1.187	2.2429
15	1.487	2.46823
20	1.738	2.62421
25	1.986	2.7576
30	2.207	2.86311
35	2.177	2.84942
40	2.19	2.85537

(6) TYESB

Time	OD	$\ln(OD/OD_0)$
0	0.126	0
5	0.252	0.69315
10	0.99	2.06142
15	1.391	2.4015
20	1.49	2.47025
25	1.69	2.5962
30	1.697	2.60034
35	1.694	2.59857
40	1.595	2.53835

(7) CBCM

Time	OD	$\ln(OD/OD_0)$
0	0.126	0
5	0.126	0
10	0.796	1.84332
15	1	2.07147
20	1.092	2.15948
25	1.194	2.24878
30	1.191	2.24627
35	1.197	2.25129
40	1.197	2.25129

(8) MAOB.

Time	OD	$\ln(OD/OD_0)$
0	0.126	0
5	0.126	0
10	0.34	0.99266
15	0.439	1.24822
20	0.446	1.26404
25	0.486	1.34993
30	0.49	1.35812
35	0.439	1.24822
40	0.446	1.26404

Table S2. Resulting parameter estimates of log × hyp fit to *Flavobacterium psychrophilum* grown on eight liquid mediums: (1) TYES, (2) Shieh, (3) modified Cytophaga, (4) Cy7, (5) FLPB, (6) TYESB, (7) CBCM and (8) MAOB.

	Parameter			
Dataset	x_0	x_m	μ	λ
1	2.7E-06	1.6	0.936	13.6
2	3.5E-05	1.4	0.807	11.9
3	2.0E-07	1.3	1.13	12.9
4	6.8E-08	0.6	0.875	19.3
5	0.04	2.5	0.850	3.0
6	0.01	1.7	1.04	4.2
7	0.02	1.2	0.524	29.4
8	0.06	0.5	0.312	37.5

Table S3. Resulting parameter estimates of log × exp fit to *Flavobacterium psychrophilum* grown on eight liquid mediums: (1) TYES, (2) Shieh, (3) modified Cytophaga, (4) Cy7, (5) FLPB, (6) TYESB, (7) CBCM and (8) MAOB.

	Parameter			
Dataset	x_0	x_m	μ	ρ
1	2.4E-04	1.4	0.369	0.0037
2	8.5E-06	1.4	0.840	0.0474
3	2.6E-05	1.3	0.483	0.0092
4	1.3E-05	0.63	0.377	0.0042
5	0.09	3.1	0.454	0.0997
6	0.01	1.8	0.856	0.1112
7	7.7E-05	1.3	1.58	0.1661
8	0.07	0.5	0.261	0.0005

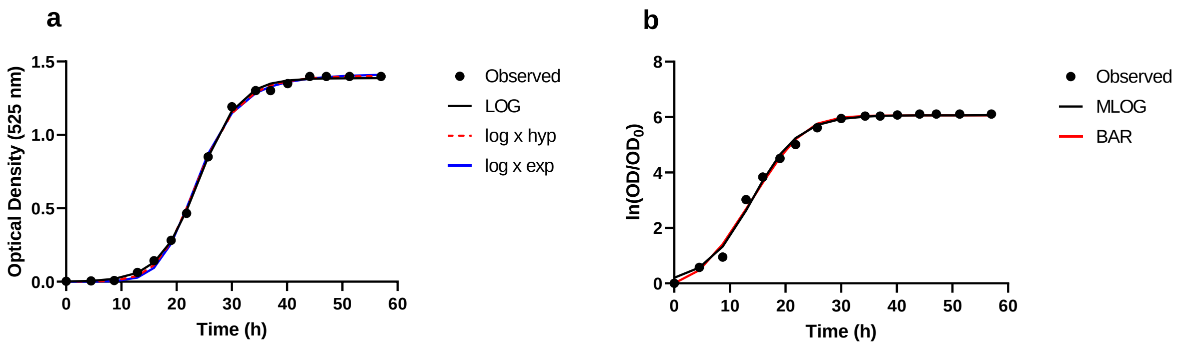

Figure S1. Optical density growth data and model predictions for *Flavobacterium psychrophilum*.

Growth predictions using (a) LOG, log × hyp, log × exp (b) MLOG and BAR grown on a Sheih liquid medium

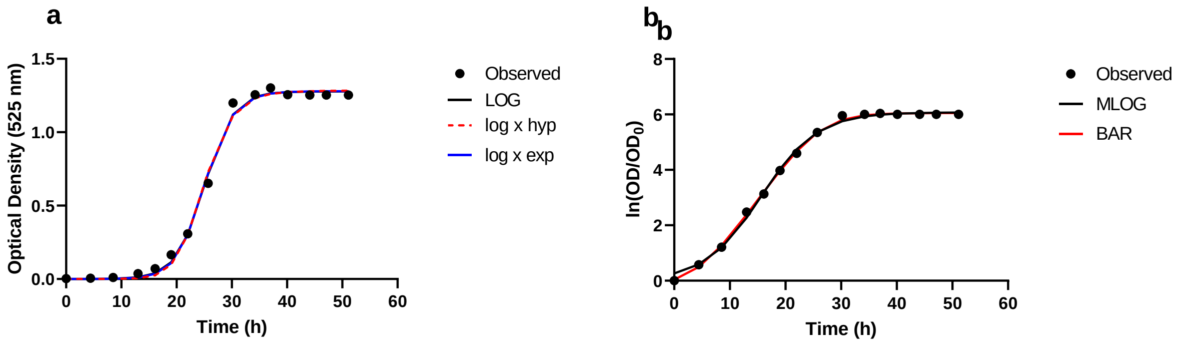

Figure S2. Optical density growth data and model predictions for *Flavobacterium psychrophilum*.
Growth predictions using (a) LOG, log × hyp, log × exp (b) MLOG and BAR grown on a modified Cytophaga liquid medium.

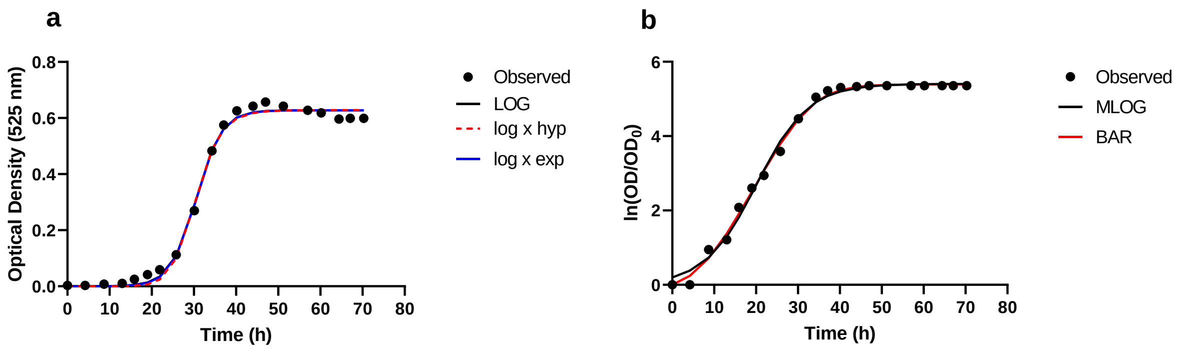

Figure S3. Optical density growth data and model predictions for *Flavobacterium psychrophilum*. Growth predictions using (a) LOG, log × hyp, log × exp (b) MLOG and BAR grown on a Cy7 liquid medium.

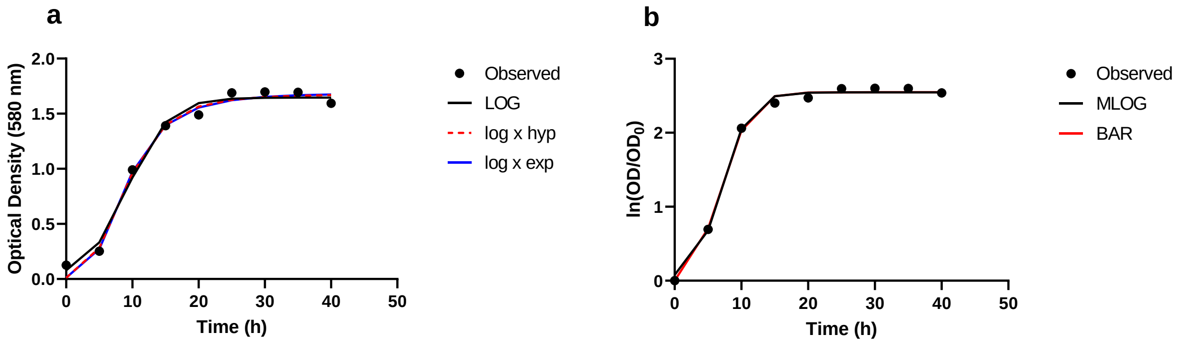

Figure S4. Optical density growth data and model predictions for *Flavobacterium psychrophilum*. Growth predictions using (a) LOG, log × hyp, log × exp (b) MLOG and BAR grown on a TYESB liquid medium.

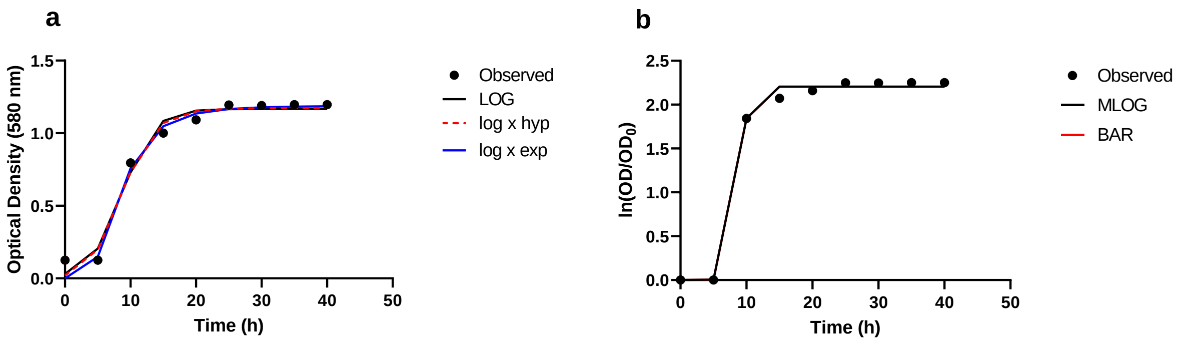

Figure S5. Optical density growth data and model predictions for *Flavobacterium psychrophilum*. Growth predictions using (a) LOG, log × hyp, log × exp (b) MLOG and BAR grown on a CBCM liquid medium.

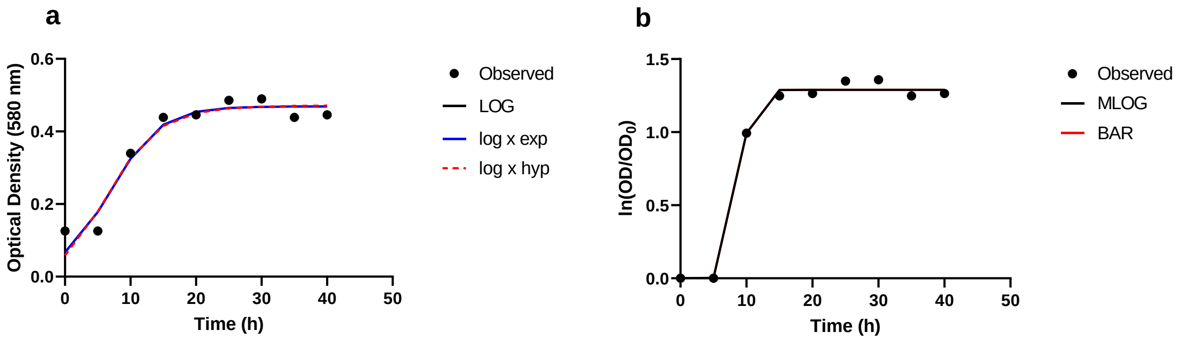

Figure S6. Optical density growth data and model predictions for *Flavobacterium psychrophilum*. Growth predictions using (a) LOG, log × hyp, log × exp (b) MLOG and BAR grown on a MAOB liquid medium.
